# The significance of triple-capsid-mutant AAV8 for treatment of Sanfilippo Syndrome Type B

**DOI:** 10.46439/stemcell.3.013

**Published:** 2022

**Authors:** Frederick Ashby, Coy Heldermon

**Affiliations:** University of Florida, College of Medicine, Gainesville, FL 32610-0278, USA

**Keywords:** Sanfilippo Syndrome, Mucopolysaccharidosis, Adeno-associated virus, Gene-therapy, AAV8, Capsid mutants

## Abstract

Sanfilippo Syndrome Type-B remains an untreatable childhood neurodegenerative disease with great burden for both patient and caregiver. Very few clinical trials have been undertaken to treat the disease, and none of these have yet yielded clinically obtainable products for patients. Caused by a simple enzyme function deficiency, Sanfilippo Syndrome Type-B has been considered a great prospect for gene-therapy interventions. Adeno-associated virus (AAV) remains a major choice for therapeutic gene delivery due to its relatively low-immunogenicity, versatility and tissue tropism. However, many clinical trials with AAV continue to use wild-type capsids, which in many cases are not able to reach stable transgene expression for long enough to be clinically effective in most cases. Previous research in AAV gene-therapy has created a litany of novel AAV capsids that can improve overall transduction efficiency far above that of wild-type AAV capsids. One such example is the triple-capsid mutant AAV8 (TCM8), which has been shown to exhibit transgene expression far superior to other capsids in Sanfilippo mouse models, specifically. Originally designed to bypass capsid ubiquitination intracellularly, mouse studies suggest this TCM8 vector outperforms both AAV5 and AAV9 when delivered to the central nervous system. This implies it as an ideal contender for an effective gene-therapy clinical trial candidate and has the potential to advance the progress of Sanfilippo Syndrome treatment. Here we provide commentary on the TCM8 vector and its context in the field of Sanfilippo Syndrome Type-B research.

## Sanfilippo Syndrome Type-B

Sanfilippo Syndrome, or Mucopolysaccharidosis (MPS) III, was first described in 1963 as an inherited condition of intellectual disability associated with significant mucopolysacchariduria, specifically of heparan sulfate [1]. The pathological elevation in mucopolysaccharides placed it in the same disease category as Hurler’s Syndrome (MPS I) and Hunter’s Syndrome (MPS II), however typically with less somatic manifestations and more central nervous system (CNS) dysfunction. MPS III is characterized on the cellular level by lysosomal distention, and grossly by organomegaly, coarsened facies and central nervous system degeneration [2]. Symptom onset typically happens in the first few years of life and can initially present as regression of developmental milestones and can be initially mistaken for isolated autism spectrum disorder (ASD). Recurrent ear, nose and throat infections are common in this disease, along with diarrhea and in some cases hearing loss [3]. After this initial phase, the next decade is typically characterized by behavioral problems, sleep disturbances and progressive cognitive and motor decline [4–8]. The final stage of the disease is typically characterized by severe CNS dysfunction, with seizures and coma [9,10]. MPS III Type A and B comprise over 80% of cases and have the most aggressive symptoms within the Sanfilippo Syndrome category with death commonly occurring within the first two decades of life. The most common cause of mortality is pneumonia, followed by cardiorespiratory failure [11].

The accumulation of heparan sulfate (HS), a sulfonated repeating-disaccharide glycosaminoglycan (GAG), in the context of MPS III occurs definitively from biallelic mutations affecting an enzyme involved in the degradation pathway. While each type is almost clinically indistinguishable, biochemically MPS III mutations affecting the *SGSH* gene (17q25.3) are categorized as Type A; while mutations affecting the *NAGLU* gene (17q21.2) are categorized as type B; mutations affecting the *HGSNAT* gene (8p11.21-p11.1) are Type C; and mutations affecting the *GNS* gene (12q14.3) are Type D[12] – with Type A and B commonly being the most severe. Of note, there remains an *ARSG* gene (17q24.2) in the HS degradation pathway, which if mutated would be referred to as Type E, however this has yet only been described in animal models [13]. All four types of confirmed human MPS III (A, B, C and D) have had causative mutations such as missense, nonsense and splicing along with small and large indels [13,14], illustrating the allelic heterogeneity of the disease. While promoter mutations and other gene-regulation-level mutations are not frequently described in MPS, promoter/3’-UTR mutations have been reported in MPS I [15]. Phenotypically, MPS III has a very wide range of severity depending on the degree of lost degradation function, due to either loss of respective enzyme expression, enzyme function or possibly a combination in rare circumstances. Due to this very simple pathophysiology, most MPS III therapies have the goal of increasing activity levels of the affected enzyme to ameliorate disease course. Enzyme Replacement Therapy (ERT) has been a reasonable proposal for treatment, along with gene-therapy and stem cell therapy, yet there are still no approved treatments for MPS IIIB.

## AAV Gene Therapy

Adeno-associated Virus (AAV), a replication deficient parvovirus, originally discovered as a contaminant [17,18], remains a popular choice for gene-therapy, in large part due to its relatively low immunogenicity, selective tissue tropism and overall versatility [19–21]. Seroprevalence studies suggest that over 90% of participants have been exposed to at least one serotype of AAV, with heavy variation between serotypes and populations [22–24]. Despite high seroprevalence, to date there have been no known diseases confirmed to be caused by the virus. However, there remains a long-standing debate on the relationship with AAV (wild or vector) and certain cancers [25,26]. The high prevalence of neutralizing antibodies to AAV in the general population remains a significant challenge to effective AAV treatments. Currently, there are three approved AAV gene-therapies (Table 1): AAV1-LPL^S447X^ driven by cytomegalovirus (CMV) promoter for treatment of Lipoprotein Lipase Deficiency (LPLD) in 2012 [27]; AAV2-RPE65 driven by a CMV/Chicken-β-actin (CβA) hybrid promoter for treatment of inherited retinal dystrophy (RD) caused by biallelic RPE65 dysfunction in 2017 [28–30]; and self-complementary AAV9-SMN1 driven by a CMV/CβA hybrid promoter for treatment of spinal muscular atrophy 1 (SMA1) in 2019 [31]. It is worth noting that each of these products utilizes wild-type AAV capsids. Currently, there are over a hundred active clinical trials involving AAV gene-therapy (ClinicalTrials.gov) treating a litany of diseases.

## Clinical Trials for MPS IIIB Treatment

**Figure 1:**
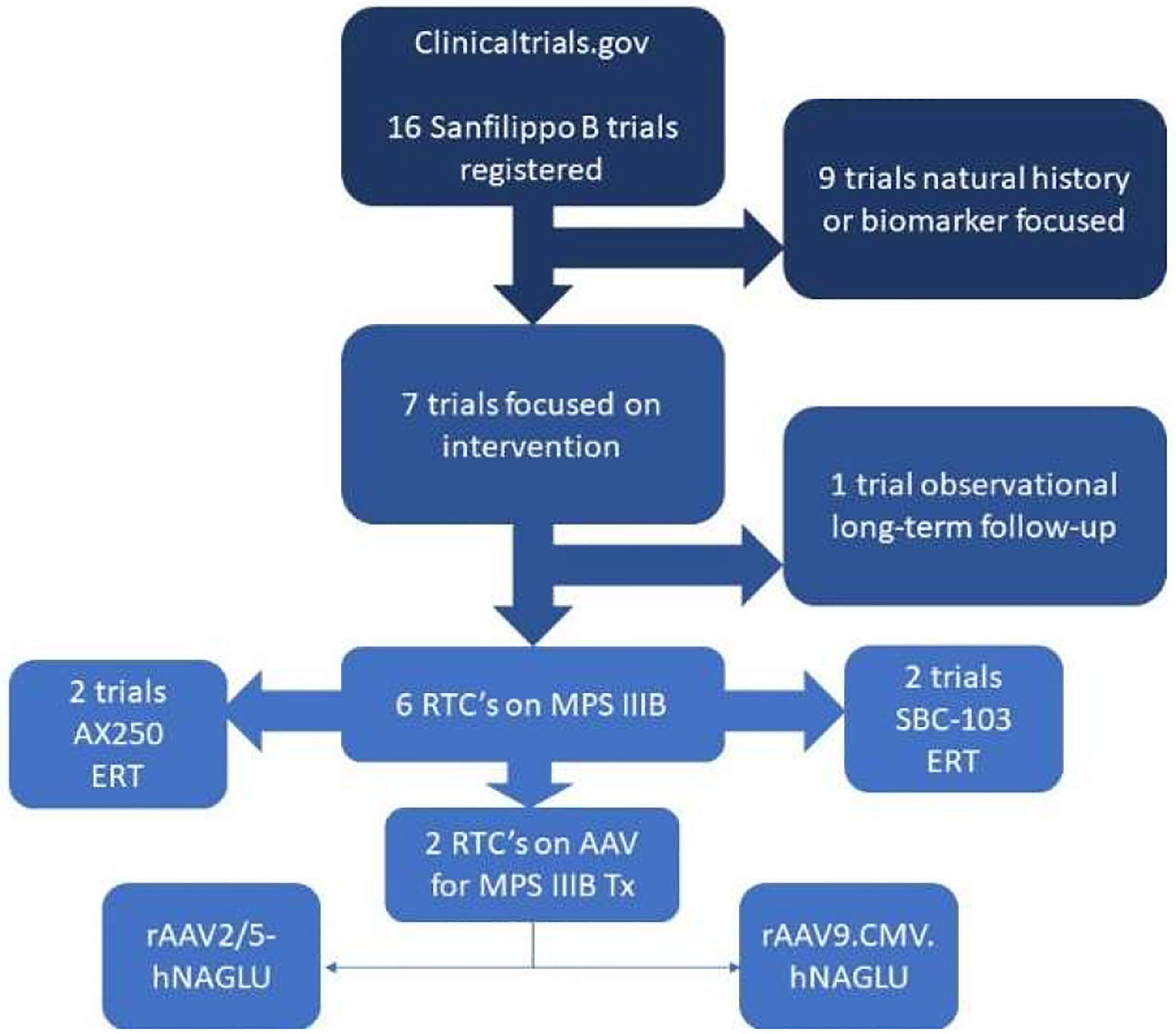
Current Clinical Trials in MPS IIIB: Out of the 16 clinical trials registered through the US National Libraries of Medicine (USNLM), 6 were randomized controlled trails (RTC’s). Only two of these trials tested rAAV treatments, and both utilized capsids from wtAAV serotypes. All data was obtained through the clinicaltrials.gov website.

The clinicaltrial.gov registry currently only has 16 trials registered for Sanfilippo B (Table 2; Figure 1). Out of these 16 trials, 9 are observational with no treatment focus. The remaining 7 were focused on novel treatments. Four of these studies investigated two candidates for ERT: AX 250 (NCT03784287; NCT02754076) and SBC-103 (NCT02324049; NCT02618512), by both intravenous (IV) and intracerebroventricular (ICV) delivery methods. AX 250 is a chimeric fusion of recombinant human α-N-acetylglucosaminidase and truncated human insulin-like growth factor 2 (rhNAGLU-IGF2), while SBC-103 is only a recombinant human α-N-acetylglucosaminidase (rhNAGLU). At least in the context of the study endpoints, such as developmental quotient (DQ), grey matter volume and age-equivalence (AE), SBC-103 did not appear to give adequate disease correction [32] when delivered IV (NCT02324049 or NCT02618512). However, it should be noted that family members of trial participants have claimed a noticeable improvement of non-endpoint metrics associated with both symptoms and quality of life, but these results have yet to be published in peer review. Meanwhile, the ICV trial of AX 250 is ongoing (NCT03784287). Two of the clinical trials for treatment of MPS IIIB investigated AAV gene therapy as a method. The first utilized a pseudotyped rAAV2/5-huNAGLU (wtAAV2 genome within a wtAAV5 capsid) administered intracerebrally (NCT03300453) by a 16-point intraparenchymal injection method in 4 patients with MPS IIIB. The patients were 20–53 months old at the time of treatment, with the youngest patient showing the best developmental outcomes overall, suggesting an optimal window for treatment [33]. However, it should be noted that a 5-year follow-up study found an escalating cell-mediated immune response in the cerebrospinal fluid (CSF) of study participants [34], underscoring the importance of recognizing the role of immunity in long-term treatment response [35]. The second AAV trial in MPS IIIB utilized rAAV9-CMVp-huNAGLU administered IV (NCT03315182). The study aimed to leverage the AAV9 serotype’s superior ability to cross the blood-brain barrier [36,37], which allows much less invasive administration. Continued development of this gene therapy product was recently discontinued by the company but had no reported significant adverse events. Additionally, the lentiviral-transduced autologous hematopoietic stem cell transplant based study that was just starting with Orchard Therapeutics was also discontinued by the company. This leaves MPS IIIB with no active trials using a gene therapy approach to curative therapy, reinforcing a need for development of approaches in this area.

## Development of the TCM8 Vector

Efforts have been made for years to improve the effectiveness of AAV capsids in the context of human gene-therapy. While relatively effective in some trials, wild-type AAV capsids have not evolved to fill the niche they serve in gene-therapy treatment, but rather have evolved to propagate and survive in their natural host environment [38]. While AAV is weakly immunogenic, systemic AAV gene-therapy typically requires very large vector loads to achieve desired effects, frequently exceeding 10^13^ vg/kg per patient. This can elicit complement activation with thrombocytopenia, humoral immunity within the liver and other organ injury, and in severe cases life-threatening anaphylactic immune responses. Improving the efficiency of AAV vector can lower the dose required to achieve clinically significant outcomes, and thus reduce the risks to the patient while reducing the production cost of treatment. Vector efficiency has previously been improved preclinically by a combination of selective evolution of capsids [39–41], site-directed mutagenesis [42,43], capsid shuffling and chimerism [44–46], and even deep-learning simulation [47].

In order to identify better serotypes and methods for transduction of the brain, AAV5, AAV8, AAV9 and AAVrh10 were compared in wild type (WT) and MPS IIIB mouse models using a variety of injection methods, IV, thalamic, ventricular, ventral tegmental area or 6 intraparenchymal sites, to deliver a green fluorescent protein (GFP) reporter. In all mice, the broadest brain transduction was with the 6 intraparenchymal site injections. In contrast, the broadest transduction of WT brain marginally favored AAV9, however AAV8 clearly provided much broader and more intense brain transduction in MPS IIIB mice, and the other serotypes were similar between WT and MPS IIIB mice [48,49].

Building upon this disease specific enhanced brain tropism, the capsid modification principle was applied to AAV8 capsids. The triple-capsid mutant AAV8 (TCM8) proposed for treatment of Sanfilippo Syndrome type-B was originally designed to address the issue of capsid ubiquitination leading to proteasomal degradation of vectors post viral entry. Proteasomal degradation of capsids has been extensively reported to be a major cause of lost vector efficiency [40–54], so the next logical step in improving vector efficiency for many researchers has been to identify ways to circumvent this process. The process of ubiquitination is partially dependent on phosphorylation of amino acid residues on the target protein, therefore site-directed mutagenesis of exterior-facing serine, threonine or tyrosine residues to a sterically similar hydrophobic amino acid, such as phenylalanine for cyclic groups or valine for aliphatic groups, was shown to profoundly inhibit the ubiquitination process [42] without altering the capsid proteins’ function. The subsequent AAV8 capsids created included a double mutant (Y447 + 733F) and triple mutant (Y447 + 733F + T494V) that were designed based on these principles using X-ray crystallography to reference the exterior facing portions of the capsid. The triple-capsid mutant AAV8 had significantly stronger transduction efficiency compared to the double-mutant or wtAAV8, as measured by green-fluorescent protein (GFP) trans-gene expression in MPS IIIB mice [55]. Therefore, it was proposed as an optimal candidate to explore as a clinical therapy vector, specifically for MPS IIIB. Subsequently this AAV TCM8 vector was made with a codon-optimized NAGLU sequence insert and used for neonatal treatment of MPS IIIB mice with either the 6 intra-parenchymal injections or a single cisterna magna injection (Publication Currently Under Review). The treated mice demonstrate broad and high-level brain NAGLU activity and complete correction of functional and lifespan measures with either injection method demonstrating the clinical potential of the AAV TCM8 vector for correction of this disorder.

## Conclusion

In summary, the results from the TCM8 studies in MPS IIIB overall suggest that this modified capsid vector may be capable of treating MPS IIIB patients with far greater efficiency than any treatments currently being investigated in humans. This is particularly apparent by the results published by Gilkes et al. 2016 [49], which suggest wtAAV8 is superior to the two other capsid serotypes currently under clinical trial (AAV5 & AAV9), at least in MPS IIIB mice. The subsequent follow-up by Gilkes et al. in 2021[55] show a profoundly increased transduction efficiency in TCM8 above wtAAV8, which would imply a commensurately higher efficiency than wtAAV5 & wtAAV9. Our follow-up studies using a therapeutic gene (Publication Currently Under Review) also show significant results. All of this is to suggest TCM8 may make an ideal candidate for clinical trial in MPS IIIB patients. One important limitation to the work done thus far relates to the phylogenetic divergence between humans and mice, and past animal studies in AAV have demonstrated the considerable effect this can have on AAV tissue tropism. One such example is the tropism of wtAAV8 for mouse hepatocytes without such a commensurate effect being observed in human hepatocytes [56], underscoring the need for humanized-liver mouse models in hepatocyte targeting rAAV studies and the consideration of humanized tissue models in-general. While it important to note that rAAV expression in a mouse model may or may not translate to humans well, currently the evidence suggests AAV TCM8 is a prime candidate for rAAV treatment of MPS IIIB. With the absence of a non-human primate MPS IIIB model, it is difficult to test TCM8 comparatively in primates preclinically, especially if this preference for TCM8 in the CNS is disease model dependent, as appears to be the case in the previous mouse studies.[49]

Given the lack of current treatments or gene therapy clinical trials in this disease, the AAV TCM8-coNAGLU gene therapy vector is highly promising to advance treatment progress for MPS IIIB patients.

## Figures and Tables

**Table 1:** Currently Approved AAV Gene-therapies.

Capsid	Brand Name	Generic Name	Disease	Trans-Gene	Clinical Trial
wtAAV1	Glybera^®^	Alipogene tiparvovec	LPLD	CMVp-*LPL*(S447X)	NCT02904772 *
wtAAV2	Luxturna^®^	voretigene neparvovec-rzyl	RD (RPE65 subtype)	CMVp/CβAp-RPE65	NCT00999609
wtAAV9	Zolgensma^®^	onasemnogene abeparvovec-xioi	SMA1	CMVp/CβAp-sc*SMN1*	NCT03381729

* Trial discontinued by company.

**Table 2:** Sanfilippo Type-B Clinical Trials Summary.

Study Name	Study Type	Study Identifier	Intervention	Study Sponsor	Results
Natural History Study of Patients With Mucopolysaccharidosis Type IIIB (MPS IIIB, Sanfilippo Syndrome Type B)	Observational, Prospective, Cohort	NCT01509768	N/A	Shire	Published in peer-review [57], completed 2013
Natural History Studies of Mucopolysaccharidosis III	Observational, Prospective, Cohort	NCT02037880	N/A	Nationwide Children’s Hospital	None provided, completed 2015
Natural History Study to Characterise the Course of Disease Progression in Participants With Mucopolysaccharidosis Type IIIB	Observational, Prospective, Case-series	NCT02293408	N/A	Alexion Pharm.	None provided, completed 2017
Neurobehavioral Phenotypes in MPS III	Observational, Prospective, Cohort	NCT01873911	N/A	University of Minnesota; NIH U54NS065768	Published in peer-review [7,8], completed 2014
A Retrospective Chart Review of Deceased Patients With Mucopolysaccharidosis Type IIIB	Observational, Retrospective, Case-series	NCT02293382	N/A	Alexion Pharm.	None provided, completed 2015
A Study of Mucopolysaccharidosis Type IIIB (MPS IIIB)	Observational, Prospective, Case-series	NCT02493998	N/A	Allievex Corp.	None provided, completed 2019
Biomarker for Sanfilippo Type A-B-C-D Disease (BioSanfilippo)	Observational, Prospective, Cohort	NCT02298686	N/A	CENTOGENE GmbH Rostock	None provided, completed 2021
Evaluation of Blood Brain Barrier Integrity and Structural Abnormalities in MPS IIIB Patients Using Multimodal Magnetic Resonance Imaging	Observational, Prospective, Case-series	NCT02090179	N/A	Alexion Pharm.	None provided, completed 2016
A Treatment Study of Mucopolysaccharidosis Type IIIB (MPS IIIB)	Interventional, Randomized Controlled Trial, Phase-1/2	NCT02754076	ERT, AX 250, IV	Allievex Corp.	None provided, completed 2020
A Treatment Extension Study of Mucopolysaccharidosis Type IIIB	Interventional, Randomized Controlled Trial, Phase-2	NCT03784287	ERT, AX 250, ICV	Allievex Corp.	None provided, ongoing until 2025
Safety, Pharmacokinetics, and Pharmacodynamics/Efficacy of SBC-103 in Mucopolysaccharidosis III, Type B (MPS IIIB)	Interventional, Randomized Controlled Trial, Phase-1/2	NCT02324049	ERT, SBC-103, ICV	Alexion Pharm.	Published on USNLM, completed 2017
A Open Label Study in Previously Studied, SBC-103 Treatment Naïve MPS IIIB Subjects to Investigate the Safety, Pharmacokinetics, and Pharmacodynamics / Efficacy of SBC-103 Administered Intravenously	Interventional, Randomized Controlled Trial, Phase-1/2	NCT02618512	ERT, SBC-103, IV	Alexion Pharm.	Published on USNLM, Published in peer-review [32], completed 2018
Intracerebral Gene Therapy in Children With Sanfilippo Type B Syndrome	Interventional, Randomized Controlled Trial, Phase-1/2	NCT03300453	Gene-therapy, rAAV2/5hNAGLU	UniQure Biopharma B.V.	Published in peer-review [33], completed 2019
Gene Transfer Clinical Trial for Mucopolysaccharidosis (MPS) IIIB (MPSIIIB)	Interventional, Randomized Controlled Trial, Phase-1/2	NCT03315182	Gene-therapy, rAAV9.CMV. hNAGLU, ABO-101	Abeona Therapeutics, Inc	None provided, ongoing until 2022
A Long-term Follow-up Study of Patients With MPS IIIB Treated With ABO-101	Observational, Prospective, Cohort	NCT04655911	Prior participation in ABO-101	Abeona Therapeutics, Inc	None provided, ongoing until 2026

ERT: Enzyme Replacement Therapy; IV: Intravenous; ICV: Intracerebroventricular
